# Supplementary material for: Identification and functional analysis of non-coding regulatory small RNA FenSr3 in Bacillus amyloliquefaciens LPB-18
Source: PeerJ. 2023 May 15;11:e15236. doi: 10.7717/peerj.15236 (PMC10194069; doi:10.7717/peerj.15236)
Supplement: Supplemental Information 4 [file peerj-11-15236-s004.zip › KO/CK-vs-T1_map/map05134.html]

KEGG PATHWAY: Legionellosis - Reference pathway


|  |  |
| --- | --- |
| **Legionellosis - Reference pathway** |  |

[
Pathway menu
| Organism menu
| Pathway entry
| Show description
| User data mapping
]

Legionellosis is a potentially fatal infectious disease caused by the bacterium Legionella pneumophila and other legionella species. Two distinct clinical and epidemiological syndromes are associated with Legionella species: Legionnaires' disease is the more severe form of the infection, which may involve pneumonia, and Pontiac fever is a milder respiratory illness.
The pathogenesis of L. pneumophila is derived from its growth within lung macrophages. One of the L. pneumophila's type IV secretion systems, the Dot/Icm secretion system, is of critical importance for its ability to replicate and to cause disease. The Dot/Icm substrates modulate multiple host cell processes and in particular, redirect trafficking of the L. pneumophila phagosome and mediate its conversion into an ER-derived organelle competent for intracellular bacterial replication. L. pneumophila also manipulates host cell death and survival pathways in a way that allows continued intracellular replication.

|  |  |  |
| --- | --- | --- |
| Reference pathway | 184% 150% 122% 100% 82% 67% 55% | 图片下载 |
